# A delta radiomics model based on ultrasound images predicts response to neoadjuvant therapy in triple negative breast cancer

**DOI:** 10.1002/acm2.70384

**Published:** 2025-11-23

**Authors:** Qiaoliang Chen, Xinyan Qin, Haiwen Du, Xiuling Ma, Shuangxiu Tan

**Affiliations:** ^1^ Department of Nuclear Medicine Nanjing Drum Tower Hospital Affiliated Hospital of Medical School, Nanjing University Nanjing China; ^2^ Medical School Nanjing University Nanjing China; ^3^ Department of Ultrasound Medicine Nanjing Drum Tower Hospital Affiliated Hospital of Medical School, Nanjing University Nanjing China

**Keywords:** neoadjuvant therapy, pathologic complete response, radiomics, triple negative breast cancer, ultrasound

## Abstract

**Background:**

Breast cancer is a common malignancy in women worldwide, with triple negative breast cancer (TNBC) being a particularly aggressive subtype. Current methods for assessing neoadjuvant therapy (NAT) response are often delayed, limiting timely adjustments to therapy. Delta radiomics offers a promising non‐invasive approach to predict treatment outcomes by analyzing imaging changes over time.

**Methods:**

A retrospective analysis was conducted on 101 female patients with TNBC who underwent NAT. A total of 972 delta radiomic features were extracted from ultrasound images acquired both pre‐ and post‐NAT. *T*‐test and least absolute shrinkage and selection operator (LASSO) were applied to select features for delta radiomics model development. A combined model was constructed by integrating the delta radiomics model with independent predictors. Receiver operating characteristic (ROC) curves, calibration curve, and decision curve analysis (DCA) were used to assess the predictive efficacy, calibration, and net clinical benefit of the models, respectively.

**Results:**

Multivariate regression analysis revealed that change rate of size (delta size) (odds ratio [OR] 2.74; *p* = 0.003) and Adler grade (pre‐NAT) (OR 0.21; *p* = 0.030) were independent factors that influenced the prediction of pathologic complete response (pCR). Nine delta radiomics features were identified as significant and a delta radiomics model was subsequently developed. The combined model, which incorporates the delta radiomics model, delta size, and Adler grade, demonstrated an area under the curve (AUC) value of 0.850 (95% confidence interval [CI] 0.752–0.947) in the training cohort and 0.787 (95% CI 0.588–0.986) in the validation cohort. The calibration curves demonstrated that the combined model exhibited high calibration. The DCA showed a substantial net benefit across a range of clinically relevant risk thresholds.

**Conclusions:**

The delta radiomics model based on ultrasound images has good predictive value for predicting pCR after NAT in TNBC and has the potential for clinical application.

## 1 INTRODUCTION

Breast cancer is among the most prevalent malignant neoplasms afflicting women globally, with its incidence exhibiting a marked increase with advancing age.^1^ Triple negative breast cancer (TNBC) constitutes 24.0% of newly diagnosed breast tumors.^2^ The efficacy of endocrine therapy and targeted therapy is diminished due to the negative expression of estrogen receptor (ER), progesterone receptor (PR), and human epidermal growth factor receptor 2 (HER2) in TNBC. TNBC is frequently characterized by high treatment resistance, high recurrence and metastasis rates, and poor prognosis.^3^, ^4^ Neoadjuvant therapy (NAT) constitutes a critical component of the TNBC treatment strategy, with the dual objectives of reducing tumor volume to enhance surgical feasibility and facilitating the development of personalized treatment regimens through comprehensive evaluation of therapeutic efficacy.^5^, ^6^ The implementation of NAT has been demonstrated to result in a highly favorable pathologic complete response (pCR), a development that significantly impacts the long‐term prognosis of patients.^7^ Presently, the clinical routine depends on postoperative pathology assessments to ascertain the efficacy of the system. This approach is characterized by a significant delay, impeding the ability to make necessary adjustments to treatment decisions.

Radiomics, a term that denotes the extraction of quantitative features from medical images, has emerged as a novel technological avenue for real‐time assessment and precise prediction of tumor treatment responsiveness. This approach boasts significant advantages, including non‐invasiveness and reproducibility, thereby underscoring its broad application prospects.^8^, ^9^, ^10^ A number of studies have indicated that radiomics models have the capacity to predict the efficacy of NAT in cases of TNBC.^11^, ^12^ TNBC is a complex and heterogeneous tumor that necessitates meticulous and continuous observation at each stage of the NAT process.^2^ Delta radiomics is predicated on the fundamental principle of comparing medical imaging data at disparate time points to identify the characteristics of imaging changes before and after treatment, thereby unveiling the tumor's response to its dynamic evolution.^13^, ^14^ In comparison with conventional static time‐point imaging analysis methodologies, the primary benefit of delta radiomics lies in its capacity to discern temporal alterations within the tumor microenvironment in response to therapeutic interventions, thereby facilitating more precise treatment outcome assessment.^15^, ^16^

The objective of this study was to develop a delta radiomics model based on pre‐ and post‐NAT ultrasound images of patients with TNBC, as well as to investigate its predictive value for pCR after NAT.

## 2 MATERIALS AND METHODS

### 2.1 Study subjects

**FIGURE 1:**
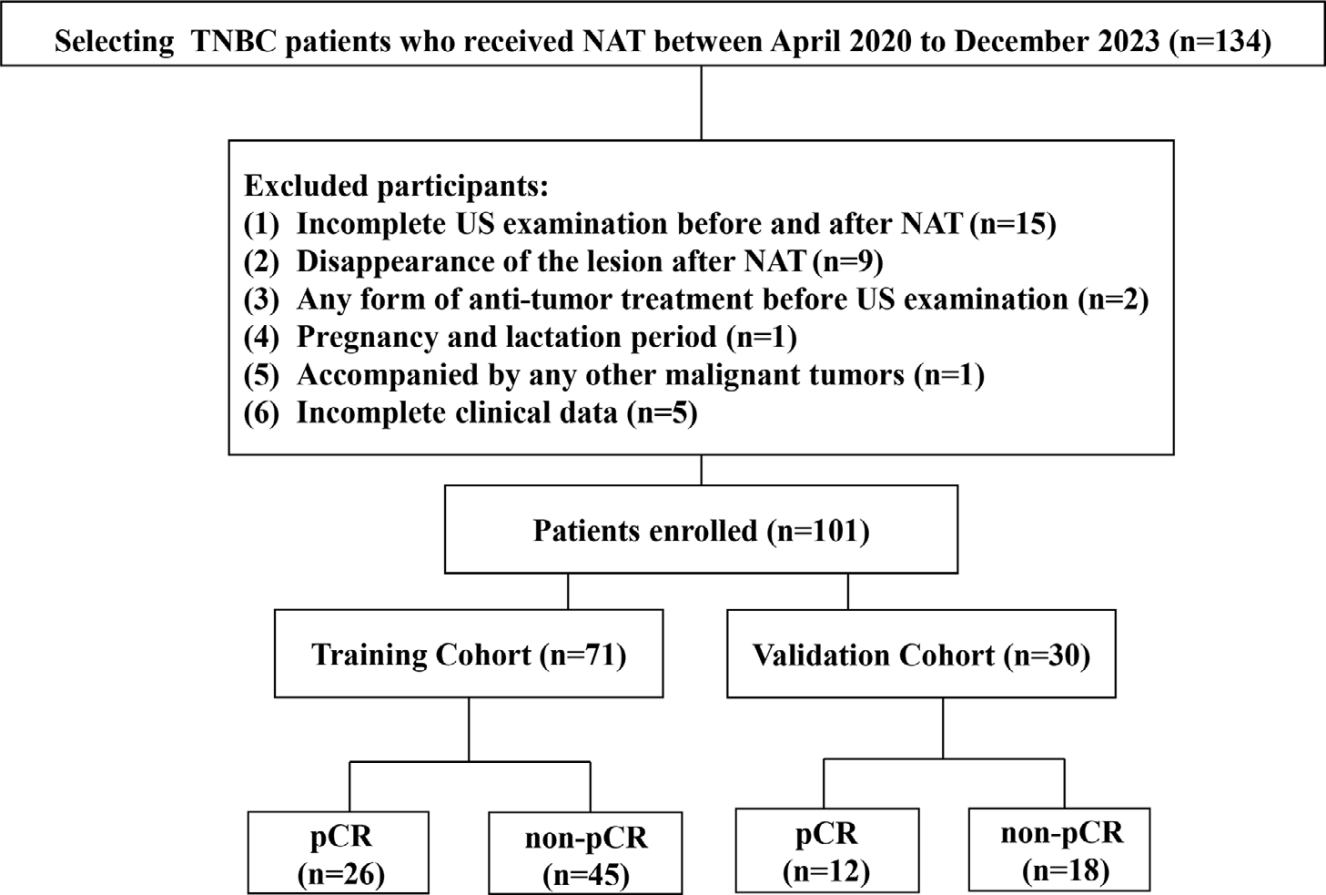
Flowchart shows study population and exclusion criteria. Pathologic complete response.

A total of 101 female patients diagnosed with TNBC between April 2020 and December 2023 were collected. The diagnostic criteria for TNBC are as follows^2^: nuclear staining of tumor cells ≤ 1% for both ER and PR, and HER2‐negative (immunohistochemistry scores of 0, 1+, or 2+ and negative fluorescence in situ hybridization). Inclusion criteria (1) diagnosed as breast cancer by pathology and TNBC by immunohistochemistry; (2) ultrasonography was performed and was able to detect breast lesions with satisfactory image quality. Exclusion criteria (1) absence of pre‐NAT or post‐NAT ultrasound examinations; (2) disappearance of the tumor as shown by post‐NAT ultrasound; (3) any form of antitumor therapy prior to the first ultrasound examination; (4) combination of pregnancy or breastfeeding; (5) combination of other malignant tumors; and (6) incomplete clinical data. The NAT regimen was a combination of docetaxel, epirubicin, and cyclophosphamide for six to eight cycles. The pathological assessment was generally completed within 3 weeks after the conclusion of NAT. Patients were adjudged to have achieved pCR based on postoperative pathology. The samples were then separated into training (*n* = 71) and validation (*n* = 30) cohorts according to a ratio of 7:3. The pCR and non‐pCR cohorts consisted of 26 (36.6%) and 45 (63.4%) cases in the training set, and 12 (40.0%) and 18 (60.0%) cases in the validation set, respectively. As Figure 1 illustrates, the study population and exclusion criteria were delineated. This study was approved by the institutional ethics committee of our institution. Informed consent was obtained from all patients, and the requirement for signed documentation was waived.

### 2.2 Collection of clinical and ultrasound data

The Mindray Resona 7 (Shenzhen, China), GE Logiq E9 (Boston, USA), and Philips Epiq7 (Amsterdam, the Netherlands) ultrasound imagers (line array probe frequency 4.0–14.0 MHz) were utilized to perform ultrasound examinations on the patients. Prior to NAT, routine ultrasound features, including lesion location, size, morphology, borders, calcification, and Adler grade (pre‐NAT), were systematically documented. Additionally, the breast imaging reporting and data system (BI‐RADS) grade was evaluated to ensure a comprehensive assessment of the breast imaging findings. All patients underwent ultrasound review at the end of NAT cycle 2, and the tumor size (post‐NAT size) was recorded. Change rate of size (delta size) = (pre‐NAT size—post‐NAT size)/ pre‐NAT size × 100%. Tumor sizes were independently performed and averaged by two physicians.

### 2.3 Radiomics feature extraction and construction of radiomics signature

Prior to the segmentation process, all ultrasound images were standardized. All images were uniformly resampled to an identical spatial resolution (1 × 1 mm), and histogram‐based gray‐level normalization was applied to reduce inter‐vendor contrast differences. The employed resampling and normalization techniques were anticipated to enhance the robustness of the radiomic features to variations in acquisition protocols across different scanners; nevertheless, future external validation is warranted to confirm this. The 3D Slicer 5.6.2 software was utilized to segment the region of interests (ROIs) of the largest cross section of TNBC lesions. Physician A performed the segmentation, blinded to the results, and 30% of the sample was randomly selected for segmentation by physician B. The Dice coefficient was used to evaluate the reliability of delineation by two doctors. An average Dice similarity coefficient greater than 0.9 was considered to demonstrate good repeatability. The pyradiomics package was used for extracting radiomics features from the pre‐ and post‐NAT ultrasound images. Delta radiomics features are defined as the difference between post‐NAT and pre‐NAT features. Variables with a variance of 0 were removed, and the remaining variables were *Z*‐score normalized. The *t*‐test was used to retain the features with differences between groups (*p* < 0.05). Feature selection was performed using least absolute shrinkage and selection operator (LASSO) regression with 10‐fold cross‐validation. The lambda (*λ*) value corresponding to the minimum standard error was selected. All variables with non‐zero coefficients in this model were retained as the final features. Using logistic regression (LR), a delta‐radiomics model was constructed with the selected features.

### 2.4 Construction and evaluation of the model

Univariate and multivariate analyses were used to identify independent predictors of clinical ultrasound characteristics that predict pCR of TNBC after NAT. A combined model was constructed by integrating the delta radiomics model with independent predictors. These models are combined into a nomogram. The predictive performance of each model was evaluated using the area under the ROC curve (AUC). Furthermore, the optimal cutoff threshold for each model was determined by the Youden index, and subsequently used to calculate accuracy, sensitivity, specificity, positive predictive value (PPV), negative predictive value (NPV), and F1_score. The Hosmer–Lemeshow test was employed to assess model calibration. The accuracy of the combined model was assessed by calibration curves. Decision curve analysis (DCA) was performed to assess the net clinical benefit of each model.

### 2.5 Statistical analysis

Analyses were conducted using Python 3.9, R 4.3, and SPSS 27.0. The normality of the data was assessed through the implementation of the Shapiro–Wilk test. Continuous data used the nonparametric Mann–Whitney *U* test. Count data used the Pearson Chi‐square test. The ROC curves were plotted utilizing the “ROCR” package (R library). The DCA was applied with the “rms” package (R library). Differences were considered statistically significant at *p* < 0.05.

## 3 RESULTS

### 3.1 Patient characteristics

A comparison of the training and validation cohort did not reveal differentiating features (Table 1). A subsequent between‐group comparison of the training cohort revealed statistically significant differences in post‐NAT size (*p* < 0.001), delta size (*p* < 0.001), and Adler grade (*p* = 0.018) between the pCR cohort and non‐pCR cohort (Table 2). Univariate and multivariate regression analysis revealed that delta size (odds ratio [OR] 2.74; 95% confidence interval [CI] 1.40–5.37; *p* = 0.003) and Adler grade (OR 0.21; 95% CI 0.05–0.86; *p* = 0.030) were independent factors that influenced the prediction of pCR (Table 3).

**TABLE 1:** Comparison of clinical‐US characteristics between the training and validation cohort.

Characteristics	Total (*n* = 101)	Training cohort (*n* = 71)	Validation Cohort (*n* = 30)	*p*
Age/Year, M (Q_1_, Q_3_)	53.0 (41.0, 59.0)	52.0 (44.0, 57.0)	56.00 (41.0, 64.5)	0.324
Pre‐NAT size/cm, M (Q_1_, Q_3_)	2.8 (2.3, 3.4)	2.80 (2.3, 3.45)	2.6 (2.4, 3.3)	0.973
Post‐NAT size/cm, M (Q_1_, Q_3_)	1.8 (1.3, 2.5)	1.8 (1.3, 2.5)	1.9 (1.3, 2.7)	0.696
Delta size/cm, M (Q_1_, Q_3_)	0.32 (0.17, 0.48)	0.35 (0.16, 0.48)	0.31 (0.19, 0.49)	1.000
Location				0.683
Left	57 (56.44)	41 (57.75)	16 (53.33)	
Right	44 (43.56)	30 (42.25)	14 (46.67)	
Boundary				0.474
Clear	12 (11.88)	10 (14.08)	2 (6.67)	
Non‐clear	89 (88.12)	61 (85.92)	28 (93.33)	
Morphology				0.965
Regular	12 (11.88)	9 (12.68)	3 (10.00)	
Non‐regular	89 (88.12)	62 (87.32)	27 (90.00)	
Calcification				0.753
Negative	65 (64.36)	45 (63.38)	20 (66.67)	
Positive	36 (35.64)	26 (36.62)	10 (33.33)	
Adler grade				0.397
0–I	70 (69.31)	51 (71.83)	19 (63.33)	
II–III	31 (30.69)	20 (28.17)	11 (36.67)	
BI‐RADS				0.238
4A	17 (16.83)	14 (19.72)	3 (10.00)	
4B	25 (24.75)	19 (26.76)	6 (20.00)	
4C	33 (32.67)	19 (26.76)	14 (46.67)	
5	26 (25.74)	19 (26.76)	7 (23.33)	

Abbreviations: BI‐RADS, breast imaging reporting and data system; NAT, neoadjuvant therapy.

**TABLE 2:** Comparison of clinical‐US characteristics between the pCR and non‐pCR cohort in the training cohort.

Characteristic	Total (*n* = 71)	pCR Cohort (*n* = 26)	non‐pCR Cohort (*n* = 45)	*p*
Age/Year, M (Q_1_, Q_3_)	52.0 (44.0, 57.0)	51.0 (44.5, 56.0)	53.00 (41.0, 58.0)	0.867
Pre‐NAT size/cm, M (Q_1_, Q_3_)	2.8 (2.3, 3.5)	2.5 (2.2, 3.2)	3.0 (2.4, 3.6)	0.201
Post‐NAT size/cm, M (Q_1_, Q_3_)	1.8 (1.3, 2.5)	1.3 (1.1, 1.7)	2.0 (1.6, 2.7)	<.001
Delta size/cm, M (Q_1_, Q_3_)	0.35 (0.16, 0.48)	0.44 (0.32, 0.56)	0.26 (0.00, 0.42)	<.001
Location				0.136
Left	41 (57.75)	18 (69.23)	23 (51.11)	
Right	30 (42.25)	8 (30.77)	22 (48.89)	
Boundary				0.553
Clear	10 (14.08)	5 (19.23)	5 (11.11)	
Non‐clear	61 (85.92)	21 (80.77)	40 (88.89)	
Morphology				0.880
Regular	9 (12.68)	4 (15.38)	5 (11.11)	
Non‐regular	62 (87.32)	22 (84.62)	40 (88.89)	
Calcification				0.197
Negative	45 (63.38)	19 (73.08)	26 (57.78)	
Positive	26 (36.62)	7 (26.92)	19 (42.22)	
Adler grade				0.018
0–I	51 (71.83)	23 (88.46)	28 (62.22)	
II–III	20 (28.17)	3 (11.54)	17 (37.78)	
BI‐RADS				0.096
4A	14 (19.72)	8 (30.77)	6 (13.33)	
4B	19 (26.76)	9 (34.62)	10 (22.22)	
4C	19 (26.76)	4 (15.38)	15 (33.33)	
5	19 (26.76)	5 (19.23)	14 (31.11)	

Abbreviations: BI‐RADS, breast imaging reporting and data system; NAT, neoadjuvant therapy; pCR, pathologic complete response.

**TABLE 3:** Results of univariate and multivariate analysis of the independent predictors in the training cohort.

	Univariate analysis			Multivariate analysis		
	OR	95% CI	*p*	OR	95% CI	*p*
Post‐NAT size	1.08	0.72–1.60	0.720			
Delta size	2.77	1.42–5.38	0.003	2.74	1.40–5.37	0.003
Adler grade						
0–I	Reference			Reference		
II–III	0.21	0.06–0.82	0.025	0.21	0.05–0.86	0.030

Abbreviations: CI, confidence interval; NAT, neoadjuvant therapy; OR, odds ratio.

### 3.2 Delta radiomics model establishment

The Dice coefficient for the ROIs outlined by the two physicians was 0.970, indicating reproducibility. A total of 972 delta radiomics features were extracted. Following selection via the *t*‐test and LASSO regression, nine delta radiomics features were used to construct the comprehensive delta radiomics model. As shown in the Table S1, the feature names, coefficients, and intercept are provided, enabling the calculation of prediction scores.

### 3.3 Model performance comparison

**FIGURE 2:**
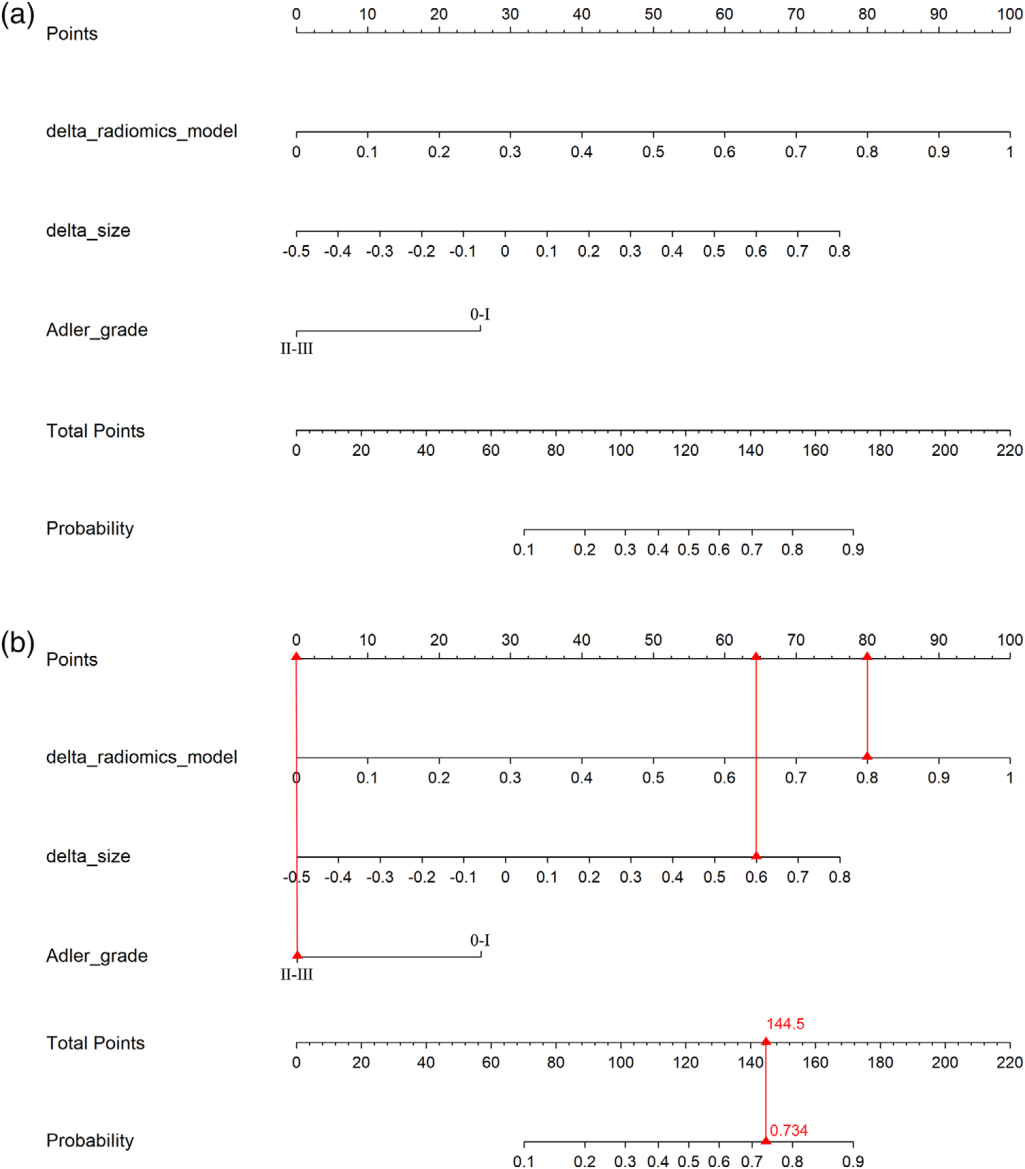
The nomogram is developed by delta radiomics model, delta size, and Adler grade (a). A worked example illustrating the clinical use of the predictive nomogram (b). This example calculates the individualized probability of pCR for a hypothetical patient. The patient's values are a delta‐radiomics score of 0.8, a delta size of 0.6, and an Adler grade of III. For each variable, locate the value on its corresponding axis and draw a line upward to the "Points" axis to determine the individual score. Sum the points from all three variables to obtain the Total Points. Locate the "Total Points" axis and draw a line straight down to the "Probability" axis. The corresponding probability for this patient is 0.734. pCR, pathologic complete response.

**FIGURE 3:**
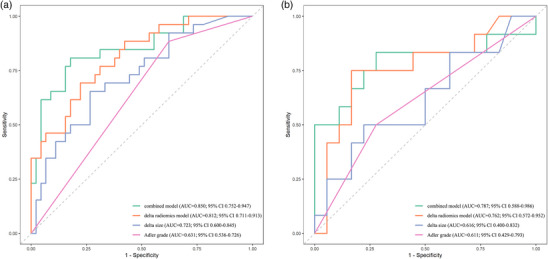
ROC curves for the combined model, delta radiomics model, delta size, and Adler grade in the training cohort (a) and the validation cohort (b). ROC, receiver operating characteristic.

**FIGURE 4:**
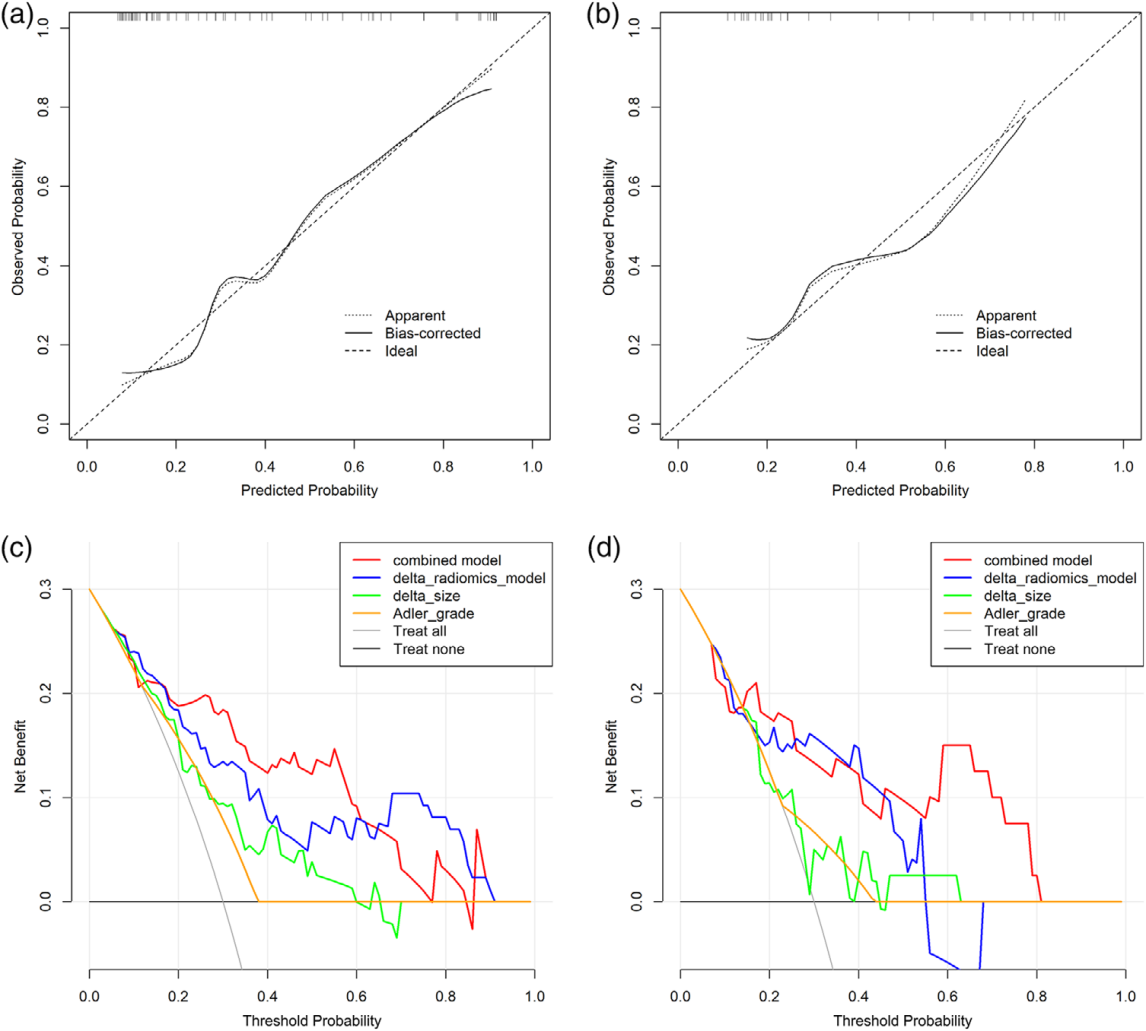
Calibration curves and DCA. The calibration curves of the combined model in the training (a) and the validation cohorts (b). DCA of the combined model, delta radiomics model, delta size, and Adler grade in the training (c) and the validation cohorts (d). DCA, decision curve analysis.

The combined model, which integrated the delta radiomics model, delta size and Adler grade was constructed and visualized as a Nomogram model (Figure 2a). To illustrate the practical clinical use of the Nomogram, a worked example for a sample patient was provided in Figure 2b. The ROC curves (Figure 3) demonstrated that the combined model exhibited an area under the curve (AUC) of 0.850 (95% CI 0.752–0.947) in the training cohort and 0.787 (95% CI 0.588–0.986) in the validation cohort. The DeLong test revealed that the joint model's AUC value exceeded the delta size (*p* = 0.021) and the Adler grade (*p* < 0.001) predicted alone. Despite the AUC value of the combined model in the training cohort not demonstrating a statistically significant increase over that of the delta radiomics model (*p* = 0.209), the combined model exhibited higher levels of accuracy, sensitivity, and specificity compared to the delta radiomics model. The efficacy of each parameter in predicting pCR was delineated in Table 4. The Hosmer–Lemeshow test indicated good fit of the combined model in both the training (*χ* ^2^ = 3.49; *p* = 0.900) and validation (*χ* ^2^ = 9.44; *p* = 0.306) cohorts. The calibration curves (Figure 4a,b) demonstrated that the combined model's actual curves in the training and validation cohorts closely resembled the ideal curves, suggesting a high degree of calibration. The DCA (Figure 4c,d) indicated that the combined model and the delta radiomics model both exhibited substantial clinical utility. The combined model demonstrated significant net benefit within threshold probability ranges of 1.1%–96.4% and 7.2%–85.9% in the training and validation cohorts, respectively.

**TABLE 4:** The performance of combined model, delta radiomics model, delta size, and Adler grade for predicting the pCR of TNBC to NAT in the training and validation cohort.

Characteristic	AUC	95% CI	Accuracy	Sensitivity	Specificity	PPV	NPV	F1 score
Training cohort								
Combined model	0.850	0.752–0.947	0.817	0.808	0.822	0.724	0.881	0.764
Delta radiomics model	0.812	0.711–0.913	0.746	0.692	0.778	0.643	0.814	0.667
Delta size	0.723	0.600–0.845	0.704	0.654	0.733	0.586	0.786	0.618
Adler grade	0.631	0.536–0.726	0.563	0.885	0.378	0.451	0.850	0.597
Validation Cohort								
Combined model	0.787	0.588–0.986	0.767	0.833	0.722	0.667	0.867	0.741
Delta radiomics model	0.762	0.572–0.952	0.800	0.750	0.833	0.750	0.833	0.750
Delta size	0.616	0.400–0.832	0.667	0.500	0.778	0.600	0.700	0.545
Adler grade	0.611	0.429–0.793	0.633	0.500	0.722	0.545	0.684	0.522

Abbreviations: AUC, area under the curve; CI, confidence interval; NAT, neoadjuvant therapy; NPV, negative predictive value, pCR, pathologic complete response; PPV, positive predictive value; TNBC, triple negative breast cancer.

## 4 DISCUSSION

This study demonstrated the predictive value of the delta radiomics model for pCR based on pre‐ and post‐NAT ultrasound images. The Nomogram model, which integrates the delta radiomics model, delta size, and Adler grade, has been demonstrated to serve as a personalized preoperative assessment tool with a high degree of accuracy.

The therapeutic options available for patients with TNBC are limited, with most treatments involving chemotherapy.^17^ The use of NAT allows for direct and early assessment of tumor biology, particularly the status of pCR, a metric that is closely associated with durable treatment response and long‐term survival.^18^ However, substantial inter‐patient variability in treatment response was observed in TNBC cases, with efficacy assessment primarily reliant on imaging and pathological features. Radiomics hold particular value in the differential diagnosis, efficacy, and prognosis prediction of breast cancer.^8^, ^19^ Yang et al. demonstrated the potential of a radiomics model based on ultrasound images to predict pCR after NAT in breast cancer.^20^

The construction principle of the delta radiomics model is predicated on the analysis of the dynamic change characteristics of tumors by comparing medical imaging data at different time points before and after treatment. This model demonstrates to effectively capture the changes in imaging characteristics caused by therapeutic interventions, thus enabling more accurate assessment of the biological response of the tumor and the therapeutic effect.^21^ In comparison with conventional single‐time point image analysis, the delta radiomics model exhibits superior sensitivity in detecting alterations in the tumor microenvironment through temporal analysis, thereby facilitating a more dynamic observation perspective for efficacy assessment.^22^ In this study, the relatively high proportion of wavelet features in the delta radiomics model suggests that higher‐order features provide more valuable information for evaluating the efficacy of neoadjuvant therapy in TNBC, better reflecting tumor heterogeneity and textural information.

A recent study reported that the radiomics model, based on dynamic contrast‐enhanced MRI examination, demonstrated a significant effect in predicting the efficacy of NAT in TNBC.^23^ A radiomics model developed from multi‐timepoint MRI during NAT in TNBC demonstrated good predictive performance in the validation cohort (AUC = 0.802). Furthermore, compared to MRI‐based models, the ultrasound‐based approach leveraged in this study offers the practical advantages of greater accessibility and lower cost, underscoring its high potential for broader clinical translation and application.^12^ Huang et al.^24^ showed that the ultrasound‐image–based delta radiomics model had a good prediction of pCR after NAT in breast cancer, with a sensitivity and specificity of 79.1% and 82.2%, respectively. This study focuses on TNBC, which was more targeted. In this study, nine optimal delta radiomics features were selected to construct a model that exhibited a superior prediction effect on pCR. The AUC values of the delta radiomics model in the training set and validation set were 0.850 (95% CI 0.752–0.947) and 0.787 (95% CI 0.588–0.986), respectively.

The Adler grade, a histopathological grading system for evaluating the malignancy of tumors, is indicative of the nutrient vascular richness of the tumor. This characteristic exerts a direct influence on the nutrient supply and metabolic activities of the tumor cells, thereby significantly impacting tumor growth and treatment response.^25^ In general, an increased vascular supply within the tumor results in greater oxygen, nutrient, and other growth factor availability for the tumor cells, thereby promoting their rapid proliferation and dissemination. This phenomenon also renders the tumor resistant to the effects of certain therapeutic drugs.^26^ As indicated in the extant literature, the rate of change of tumor size pre‐ and post‐NAT in breast cancer demonstrated to serve as a reliable predictor of pCR.^27^ Consequently, this study investigated the rate of change in tumor size pre‐NAT and post‐NAT cycle 2, and ascertained that delta size exhibited the capacity to predict pCR, which was analogous to the outcomes of a preceding study.^28^ Multivariate regression analysis revealed that delta size (OR 2.74; 95% CI 1.40–5.37; *p* = 0.003) and Adler grade (OR 0.21; 95% CI 0.05–0.86; *p* = 0.030) functioned as independent factors influencing pCR. However, the conventional assessment methods were found to have inherent limitations. The integration of a delta radiomics model within a combined model demonstrated to enhance the predictive efficacy to a significant extent. The AUC of the combined model in the training cohort exhibited a significant increase over the predictions derived from delta size (*p* = 0.021) and Adler grade (*p* < 0.001) alone.

The present study was subject to several limitations. First, this was a single‐center retrospective study, and the relatively small sample size may affect the model's generalizability and the precision of its performance estimates. This study was validated only internally, and multi‐center external validation is necessary. Secondly, manual segmentation of ROIs led to an increase in the variability of the model. Therefore, there is a need for an automated tool for the segmentation of ROIs in the future. Furthermore, as this study was not applicable to patients with no breast lesions detected by post‐NAT ultrasound, further research is necessary to determine the applicability of this study to such patients. Finally, future studies will construct more advanced machine learning models for a comparative investigation.

## 5 CONCLUSION

The combined model constructed in this study, based on the delta radiomics model and conventional ultrasound parameters, performed well in predicting the pCR of TNBC to NAT. In comparison with conventional ultrasound, the delta radiomics model exhibited enhanced diagnostic efficacy. The delta radiomics model is capable of dynamically reflecting the treatment response of TNBC to NAT and possesses the potential for application in clinical practice.

## AUTHOR CONTRIBUTIONS

Qiaoliang Chen and Shuangxiu Tan conceived and designed the project. Qiaoliang Chen, Xinyan Qin, and Haiwen Du collected the data. Qiaoliang Chen, and Xiuling Ma analyzed and interpreted the data. Qiaoliang Chen and Haiwen Du drafted the manuscript. All the authors edited and made critical revisions to the article. All authors read and approved the final manuscript.

## CONFLICT OF INTEREST STATEMENT

The authors declare no conflicts of interest.

## ETHIC STATEMENT

This study was approved by the Medical Ethics Committee of Nanjing Drum Tower Hospital, Affiliated Hospital of Medical School, Nanjing University (Approval No.: 2025‐0067‐01) and obtained the informed consent from all patients.

## Data Availability

The data that support the findings of this study are available from the corresponding author upon reasonable request.
